# The effect of oxygen concentration and temperature on nitrogenase activity in the heterocystous cyanobacterium *Fischerella* sp.

**DOI:** 10.1038/s41598-017-05715-0

**Published:** 2017-07-14

**Authors:** Lucas J. Stal

**Affiliations:** 1 NIOZ Royal Institute for Sea Research, Department of Marine Microbiology and Biogeochemistry and Utrecht University, PO Box 59, 1790 AB Den Burg, The Netherlands; 2 Department of Freshwater and Marine Ecology, Institute of Biodiversity and Ecosystem Dynamics, University of Amsterdam, Amsterdam, The Netherlands

## Abstract

Heterocysts are differentiated cells formed by some filamentous, diazotrophic (dinitrogen-fixing) cyanobacteria. The heterocyst is the site of dinitrogen fixation providing the oxygen-sensitive nitrogenase with a low-oxygen environment. The diffusion of air into the heterocyst is a compromise between the maximum influx of dinitrogen gas while oxygen is kept sufficiently low to allow nitrogenase activity. This investigation tested the hypothesis that the heterocyst is capable of controlling the influx of air. Here, the thermophilic heterocystous cyanobacterium *Fischerella* sp. was analysed for the effects of oxygen concentration and temperature on nitrogenase activity. Dark nitrogenase activity is directly related to aerobic respiration and was therefore used as a measure of the influx of oxygen into the heterocyst. Above 30% O_2_, the influx of oxygen was proportional to its external concentration. Below this concentration, the influx of oxygen was higher than expected from the external concentration. A higher or lower temperature also triggered the heterocyst to increase or decrease, respectively, dark nitrogenase activity while the external concentration of oxygen was kept constant. A higher dark nitrogenase activity requires a higher rate of respiration and therefore a higher flux of oxygen. Hence, the heterocyst of *Fischerella* sp. is capable of controlling the influx of air.

## Introduction

Nitrogenase reduces N_2_ to two molecules of ammonium - and simultaneously produces at least one molecule of hydrogen (H_2_) - at the expense of protons, electrons that are usually derived from reduced ferredoxin, and 2 ATP per electron^1^. Another property that is shared by any nitrogenase is the extreme sensitivity of the enzyme for oxygen. Nitrogenase is irreversibly inactivated by exposure to even low concentrations of O_2_^2^. Nitrogenase evolved during the pre-oxygenated state of the earth’s atmosphere^3^. With the appearance of oxygenic photosynthesis and the resulting oxygenation of the atmosphere and large parts of the earth’s biosphere, aerobic diazotrophic organisms had to evolve mechanisms to protect nitrogenase from inactivation by oxygen.

Cyanobacteria are oxygenic photoautotrophic microorganisms. Many species are capable of fixing dinitrogen but this seems enigmatic in organisms that not only thrive in aerobic environments but also evolve O_2_ as a consequence of their photosynthetic mode of life, even though photosynthesis can satisfy the demand of energy and low-potential electrons for N_2_ fixation^4^. Diazotrophic cyanobacteria evolved a variety of strategies in order to provide a micro-oxic environment for nitrogenase and allow for the incompatible processes of oxygenic photosynthesis and N_2_ fixation. One of the most elegant solutions is the differentiation of a special cell, the heterocyst, for the fixation of N_2_ in certain filamentous cyanobacteria^5^. The heterocyst differentiates from a vegetative cell at semi-regular distances along the trichome through a complex chain of events^6^.

The heterocyst develops a special glycolipid layer which serves as a gas diffusion barrier^7^. It is important to note that this glycolipid layer does not distinguish between O_2_ and N_2_^8^ and does not serve as a specific O_2_ barrier. This means that the glycolipid layer should not stop gas diffusion completely because it would then also prevent N_2_ from coming in. Hence, the glycolipid layer of the heterocyst, serving as a gas diffusion barrier, must be a compromise between the maximum influx of N_2_ and the capacity to scavenge the O_2_ by respiration or otherwise. When the capacity of scavenging O_2_ is insufficient, oxygen will accumulate in the heterocyst and nitrogenase will be inactivated. Another property of the heterocyst is that it has lost the oxygenic photosystem II but retained photosystem I. The heterocyst is therefore capable of using light as energy source but depends on the neighbouring vegetative cells for reducing equivalents, which are imported as sucrose^9–11^. The imported reducing equivalents are partly used for respiratory scavenging of O_2_ and partly used to reduce ferredoxin, which serves as the electron donor to nitrogenase^12^.

It has long been known that N_2_ fixation in the heterocyst can acclimate to environmental O_2_ concentrations^13, 14^. It is assumed that this requires an alteration of the glycolipid layer of the heterocyst to become a more or less efficient gas diffusion barrier^15^. Indeed, depending on the environment from which they originated, heterocystous cyanobacteria exhibit different nitrogenase activity characteristics that are attributed to the gas diffusion property of the glycolipid layer of the heterocyst^16, 17^. Theoretical considerations have indicated that a heterocystous cyanobacterium would maximize its daily N_2_ fixation by increasing its dark nitrogenase activity^17^. Although this might seem counter-intuitive it can be understood when considering that it would allow N_2_ fixation at any time and any place (not only in the light but also in the non-illuminated parts of the ecosystem and at night). Dark nitrogenase activity is driven by respiration. Hence, in order to maximize N_2_ fixation the heterocyst should maximize gas diffusion (to increase the influx of O_2_) to the extent of its maximum capacity to scavenge the O_2_. While the acclimation of the gas diffusion characteristics of the heterocyst glycolipid layer would be a slow process^13, 14^, a more dynamic process would represent an advantage, especially under fluctuating environmental conditions. Walsby^18^ hypothesized that the regulation of the rate of gas exchange into the heterocyst could be accomplished through the opening of the pores that connects the heterocyst with the vegetative cell, analogous to stomata in plants. The aim of this work was to test whether the heterocyst is able to acclimate the influx of gas in order to optimize nitrogen fixation.

## Results

**Figure 1:**
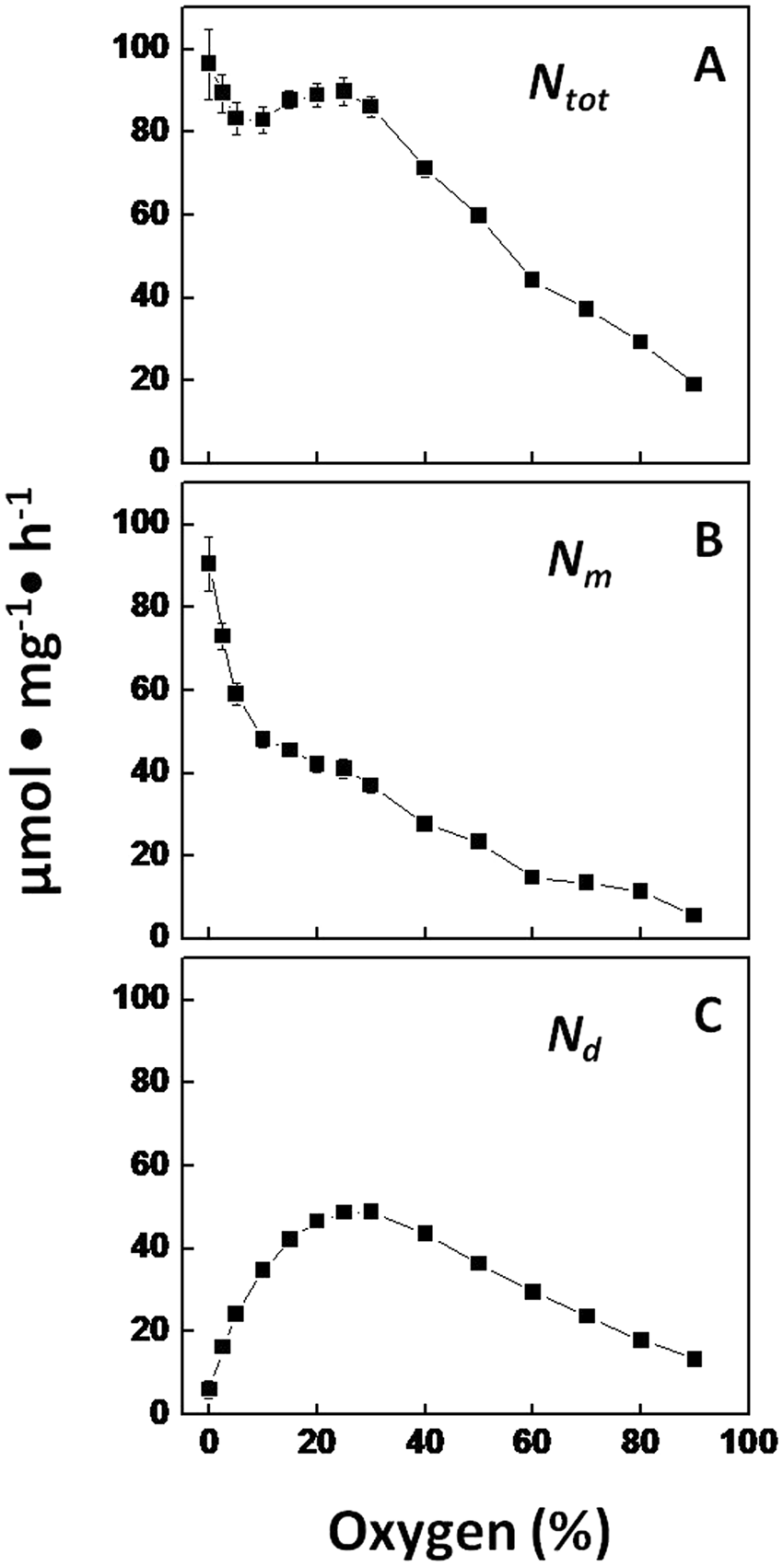
Nitrogenase activity (chlorophyll-specific rate of acetylene reduction) at different external O_2_ concentrations and a fixed temperature of 30 °C. (**A**) *N*_*tot*_, the total nitrogenase activity at light saturation; equals *N*_*m*_ + *N*_*d*_. (**B**) *N*_*m*_, the maximum light-dependent nitrogenase activity at light saturation. (**C**) *N*_*d*_, the light-independent (dark) nitrogenase activity.

The effect of O_2_ on nitrogenase activity (acetylene reduction) was tested at a fixed temperature of 30 °C. The total chlorophyll-specific nitrogenase activity (*N*_*tot*_) (the sum of the maximum light-dependent (*N*_*m*_) and light-independent (*N*_*d*_) acetylene reduction) remained constant at 88 ± 4 µmol mg^−1^ h^−1^ in the concentration range of 0–30% O_2_ (Fig. 1A). However, the relative contributions of the maximum light-dependent and light-independent fractions, respectively *N*_*m*_ (Fig. 1B) and *N*_*d*_ (Fig. 1C), changed markedly. With increasing O_2_ concentrations, *N*_*d*_ increased to its maximum at 30% O_2_, while *N*_*m*_ decreased accordingly to compensate *N*_*tot*_ to its maximum. Therefore, total nitrogenase activity (*N*_*tot*_) in the concentration range of 0–30% O_2_ was at its maximum rate at the given set of conditions. Above 30% O_2_, nitrogenase activity (*N*_*m*_ as well as *N*_*d*_) decreased linearly with O_2_ concentration (Fig. 1B,C).

**Figure 2:**
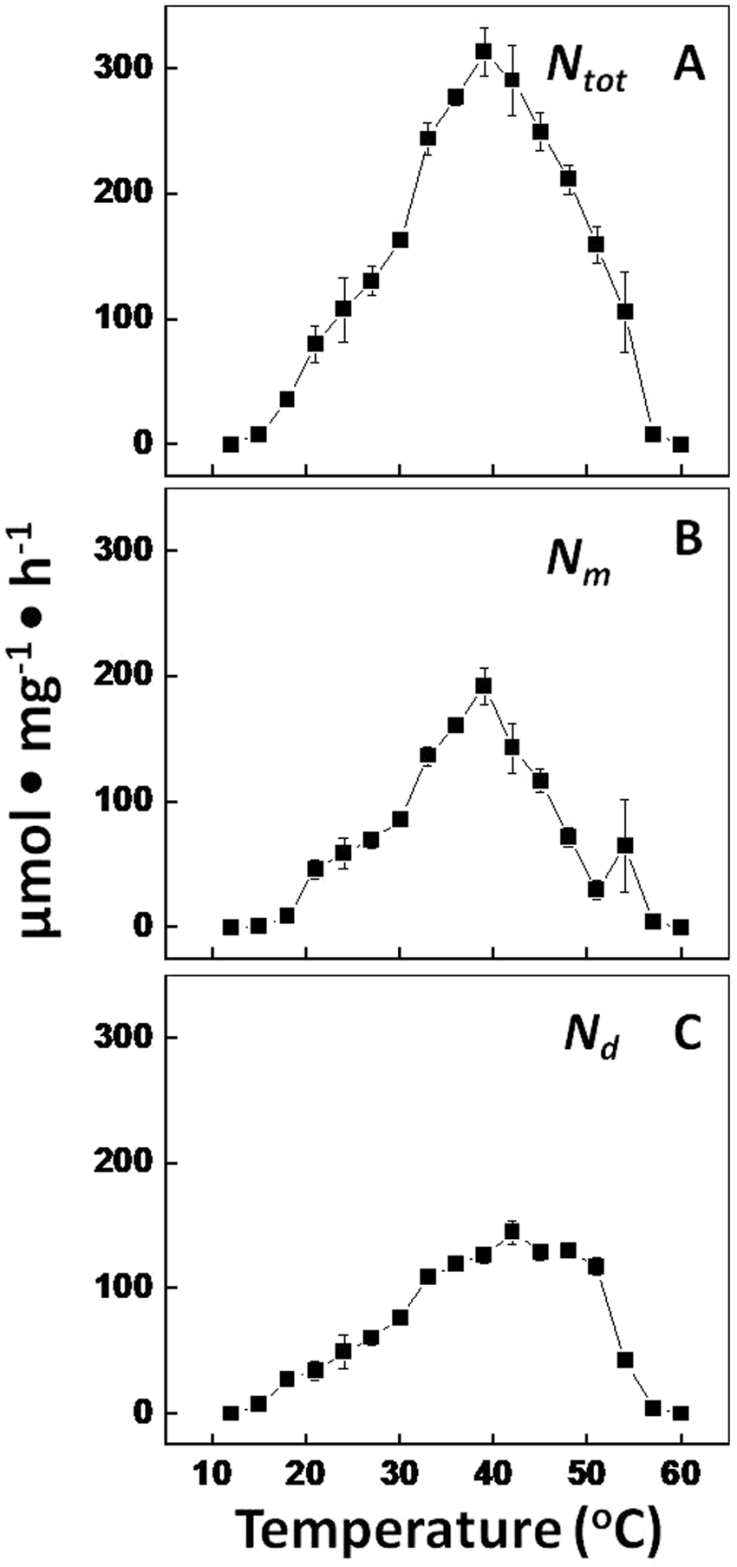
Nitrogenase activity (chlorophyll-specific rate of acetylene reduction) at different temperatures at a fixed O_2_ concentration of 20%. (**A**) *N*_*tot*_, (**B**) *N*_*m*_, (**C**) *N*_*d*_, as in Fig. 1 legend.

**Figure 3:**
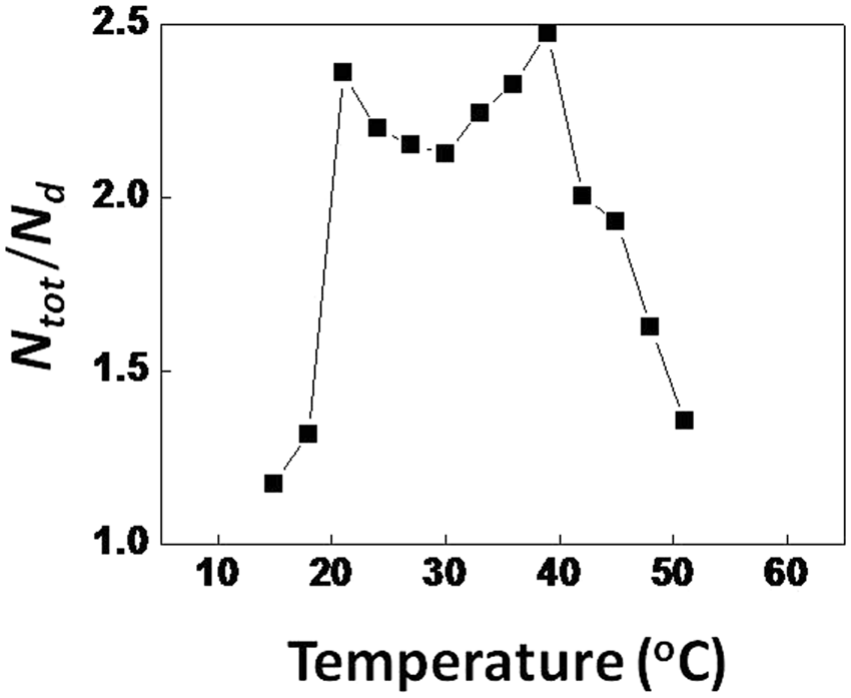
The *N*_*tot*_/*N*_*d*_ ratio at different temperatures. This ratio is dimensionless and describes nitrogenase activity in the heterocyst. The ratio indicates the contribution of photosynthesis to nitrogenase activity. A ratio of 2 means that photosynthesis and respiration contribute equally to nitrogenase activity, while a ratio of 1 means that respiration alone satisfies nitrogenase activity.

The effect of temperature on nitrogenase activity was investigated at constant O_2_ concentration of 20%. *Fischerella* is a thermophilic diazotroph allowing the investigation of a wide temperature range of 12–60 °C. The optimum temperature for nitrogenase activity in *Fischerella* was approximately 40 °C (Fig. 2). This was the case for *N*_*m*_ and *N*_*d*_ and therefore also for the total activity (*N*_*tot*_). At 12 and 60 °C there was low but still detectable nitrogenase activity (see Material and Methods for the control). Examining the *N*_*tot*_/*N*_*d*_ revealed that it remained constant in the temperature range of 21–39 °C at a value of ~2.2 ± 0.1 (Fig. 3). *N*_*tot*_/*N*_*d*_ is dimensionless and, hence, a convenient parameter to describe nitrogenase activity in the heterocyst. Below 21 °C *N*_*m*_ became almost zero and the *N*_*tot*_/*N*_*d*_ approached 1. A value of *N*_*tot*_/*N*_*d*_ of 2 means that both light-dependent and light-independent (dark) nitrogenase activity contribute equally to the total. The total nitrogenase activity increased in the temperature range 21–39 °C. Above 39 °C *N*_*m*_ rapidly declined while *N*_*d*_ remained fairly constant up to 50 °C. This resulted in *N*_*tot*_/*N*_*d*_ approaching 1, meaning that nitrogenase activity was largely driven by respiration. Above 50 °C nitrogenase activity collapsed and no meaningful analyses of *N*_*m*_ and *N*_*d*_ could be made.

## Discussion

Diazotrophic cyanobacteria are exposed to a suite of environmental factors that affect N_2_ fixation. For the fixation of dinitrogen, O_2_ concentration and temperature are particularly important and these two environmental parameters are also strongly interrelated. Temperature affects the solubility and diffusion rate of oxygen and also influences the rate of physiological (enzymatic) processes. Nitrogenase is irreversibly inactivated by O_2_ and therefore the organism has to provide an environment that is sufficiently low in O_2_ in order to be able to fix N_2_. This investigation aimed at testing the hypothesis that the heterocyst of *Fischerella* is capable of modulating the influx of gas in order to optimize N_2_ fixation. It was previously shown^17^ that natural populations of diazotrophic heterocystous cyanobacteria can improve the fixation of N_2_ best by increasing nitrogenase activity that occurs independently of light (*N*_*d*_). It should be noted that increasing dark nitrogenase activity is only possible when a higher influx and respiration of O_2_ occurs in the heterocyst. However, this must be precisely controlled so that all O_2_ that enters the heterocyst is fully and instantaneously respired in order to prevent the inactivation of nitrogenase. Detection of nitrogenase activity is a proof that this condition is fulfilled. Maximizing the influx of O_2_ into the heterocyst also maximizes the influx of N_2_, guaranteeing the highest possible fixation of N_2_.

### Interpretation of the oxygen experiment

**Figure 4:**
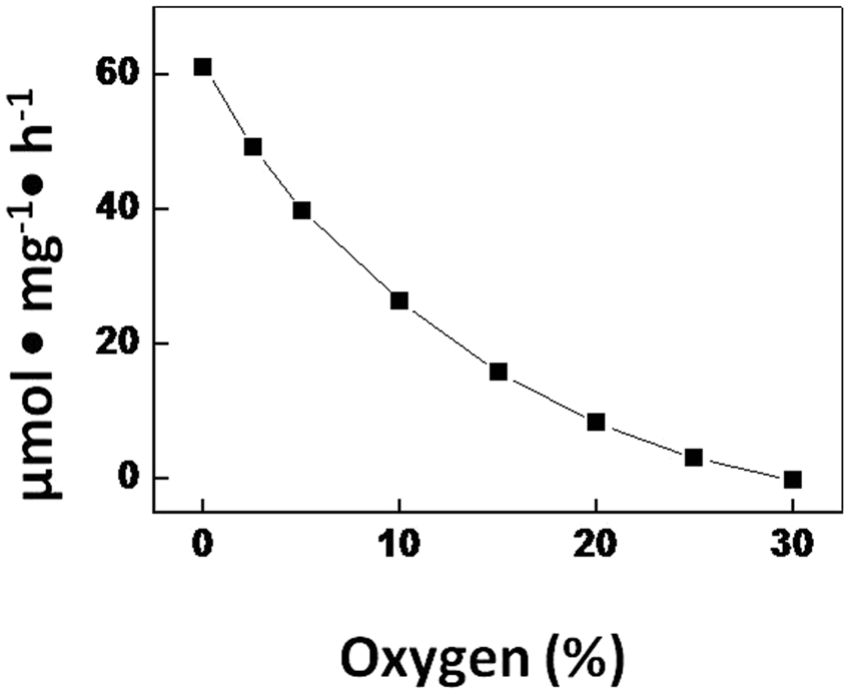
The difference between the observed *N*_*d*_ (chlorophyll-specific rate of acetylene reduction) and the values in the range 0–30% O_2_, calculated from the linear extrapolation of the linear function of *N*_*d*_ in the range 30–90% O_2_. The deviation from the linear relationship is largest at 0% O_2_, gradually decreasing to zero at 30% O_2_. The observed curve represents the variable gas flux into the heterocyst.

In the range of 0–30% O_2_ nitrogenase activity was more or less constant at 88 ± 4 µmol ethylene (mg chlorophyll *a*)^−1^ h^−1^ (Fig. 1). This must have been the maximum achievable nitrogenase activity under the given set of conditions for the following reasons. Nitrogenase activity in the range of 0–30% O_2_ was not limited by energy because *N*_*m*_ would not have decreased with increasing concentration of O_2_ and increasing *N*_*d*_. A shift in the energy source from light to respiration occurred. When energy would have been limiting an increase of *N*_*tot*_ would have been expected when O_2_ concentration increased from 0 to 30%. Nitrogenase activity in this range of O_2_ concentrations was also not limited by reducing equivalents. Otherwise *N*_*tot*_ was expected to decrease since increasing amounts of electrons are required for scavenging O_2_ when the concentration increased from 0 to 30%. Hence, the heterocyst was capable of providing all the reducing equivalents for nitrogenase and for respiring O_2_ in the range of 0–30% O_2_. The O_2_ influx into the heterocyst in this range of O_2_ concentrations as derived from the increase of *N*_*d*_ followed a dose-response relationship (Fig. 4). Likewise, the decrease of *N*_*m*_ in the range of 0–30% O_2_ could also be described by a dose-response equation. This means that the influx of O_2_ in the range of 0–30% was not simply a linear function of the external concentration.

The reason for the decrease of nitrogenase activity above 30% O_2_ is a limitation of reducing equivalents that are required for scavenging the increased influx of O_2_. The decrease in nitrogenase activity could not be attributed to an inactivation of the enzyme because controls that lowered the O_2_ concentration back to 20% restored the original activity (results not shown). The linear decrease of *N*_*d*_ above 30% O_2_ (Fig. 4) indicated that the influx (diffusion) of O_2_ was a function of the external O_2_ concentration. Assuming that the transport of reducing equivalents from the vegetative cells to the heterocysts was not limiting^19^, the linear fit of *N*_*d*_ in the range of 30–90% O_2_ gives the O_2_-specific utilization of reducing equivalents. The extrapolation of this linear fit to 0% O_2_ gives the theoretical availability of reducing equivalents for nitrogen fixation when respiration does not consume reducing equivalents (see argumentation below). The transport of sucrose into the heterocyst must be sufficient to scavenge O_2_, otherwise we would not see nitrogenase activity. It is therefore not a limiting factor.

The *N*_*d*_ data obtained in the range of 30–90% O_2_ were linearly fitted using equation (1)

1 $${\rm{y}}={\rm{ax}}+{\rm{b}}$$

where y = chlorophyll *a*-specific acetylene reduction (µmol mg^−1^ h^−1^) and x = O_2_ (%), and the constants are a = −0.61 µmol mg^−1^ h^−1^ %^−1^ and b = 67.1 µmol mg^−1^ h^−1^.

Hence, at 0% O_2_ the theoretical supply of electrons (expressed as the rate of chlorophyll-specific acetylene reduction) would equal 67.1 µmol mg^−1^ h^−1^ (because no electrons would be required to reduce O_2_), and at 100% O_2_ the theoretical residual rate of chlorophyll-specific C_2_H_2_ reduction would equal 6.1 µmol mg^−1^ h^−1^. Per %O_2_ the electron supply (expressed as the rate of chlorophyll-specific acetylene reduction) required was 0.61 µmol mg^−1^ h^−1^ ((67.1–6.1)/100%), assuming only gas diffusion through the cell envelope proportional to the external O_2_ concentration.

While the relationship of acetylene reduction and O_2_ concentration above 30% was linear and, hence, O_2_ diffusion was a function of its external concentration, below 30% O_2_ *N*_*d*_ followed a dose-response model; equation (2):

2 $${\rm{y}}={\rm{c}}+{\rm{d}}/(1+{10}^{{\rm{f}}({\rm{g}}\mbox{--}{\rm{x}})})$$

where y and x are as in equation (1). The constants c and d are −174.8 and 226 µmol mg^−1^ h^−1^, respectively. The constants f and g are 0.052%^−1^ and −11.6%, respectively.

While the diffusion through the cell envelope will still be a function of the external O_2_ concentration, an additional O_2_ influx component must be conceived in order to explain the dose response relationship. This additional component is depicted in Fig. 4 and represents the difference between the extrapolated activity calculated by equation (1) and the observed activity (Fig. 1C). It is highest at 0% O_2_, gradually decreasing to zero at 30% O_2_. The data in Fig. 4 followed a first order exponential decay model; equation (3)

3 $${\rm{y}}={\rm{h}}+{\rm{j}}{e}^{-{\rm{x}}/{\rm{k}}}$$

where y and x are as in equation (1). The constants h and j are −8.94 and 69.7 µmol mg^−1^ h^−1^, respectively, and k 14.4%.

*N*_*m*_ behaved in the same but opposite manner as *N*_*d*_. The data above 30% O_2_ were linearly fitted; equation (4):

4 $${\rm{y}}={\rm{mx}}+{\rm{n}}$$

where y and x are as in equation (1). The constants m and n are −0.5 µmol mg^−1^ h^−1^ %^−1^ and 48.4 µmol mg^−1^ h^−1^, respectively.

**Figure 5:**
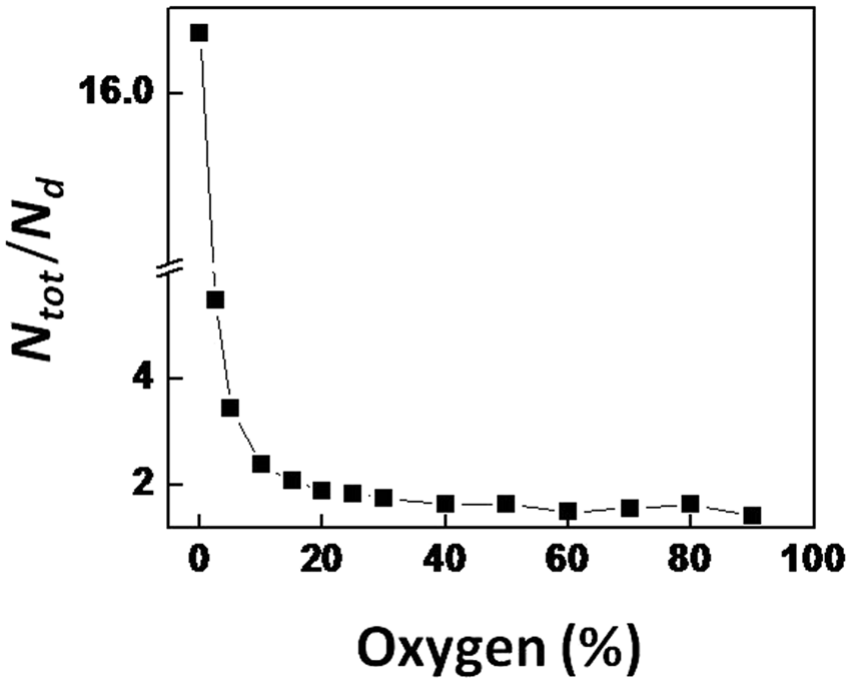
The *N*_*tot*_/*N*_*d*_ ratio at different external O_2_ concentrations. This ratio is dimensionless and describes the characteristics of nitrogenase activity in the heterocyst. The ratio indicates the contribution of photosynthesis to nitrogenase activity. Extrapolation shows that at 97% O_2_ this ratio equals 1, meaning that light is no longer required to achieve maximum nitrogenase activity. At low O_2_ concentration this ratio becomes very large because nitrogenase activity is fully driven by photosynthesis and *N*_*d*_ approaches zero.

The ratio *N*_*tot*_/*N*_*d*_ decreased with increasing O_2_ concentration indicating the increasing contribution of *N*_*d*_ to total nitrogenase activity (Fig. 5). The decrease of *N*_*tot*_/*N*_*d*_ was greatest in the range of 0–30% O_2_. *N*_*m*_ compensated for the decreasing *N*_*d*_ to the maximum nitrogenase activity in the range of 0–30% O_2_. From equation (4) it can be calculated that at 97% O_2_ *N*_*m*_ would be zero and *N*_*tot*_/*N*_*d*_ would have reached 1 meaning that respiration would provide all of the energy for nitrogenase activity.

### Interpretation of the temperature experiment

The constancy of *N*_*tot*_/*N*_*d*_ at ~2.2 in the temperature range 21–39 °C means that *N*_*m*_ and *N*_*d*_ increased in the same proportion. The value of ~2.2 for *N*_*tot*_/*N*_*d*_ is regularly observed in heterocystous cyanobacteria and may be optimal^16^. It may represent an optimal compromise between the influx of gas into the heterocyst and its capacity to scavenge O_2_. It is important to note that *N*_*d*_ can increase only when more O_2_ enters the heterocyst, allowing more respiratory energy generation. While the external concentration of O_2_ was held constant, the only possibility was that the heterocyst increased the influx of O_2_. The consequent draw on reducing equivalents for respiration resulted in a lower total nitrogenase activity, realized by a markedly lower *N*_*m*_.

In the temperature range 21–39 °C the *Q*_*10*_ could be calculated. The *Q*_*10*_ gives the increase of the rate of a process for a 10 °C rise in temperature. For biochemical processes the *Q*_*10*_ equals approximately 2, meaning a doubling for each 10 °C rise in temperature (or halving at each 10 °C decrease in temperature). For the total nitrogenase activity (*N*_*tot*_) as well as for *N*_*m*_ a value of 2.3 was calculated and 2.2 for *N*_*d*_, hence *Q*_*10*_ was well within the expected range for a biochemical process. However, if *N*_*d*_ would only depend on the rate of diffusion of O_2_ into the heterocyst the *Q*_*10*_ is expected to be only marginally above 1 (1.084), that results from the temperature effects on O_2_ solubility and diffusion constant^17^. The *Q*_*10*_ of 2.2 for *N*_*d*_ means that respiration was not limited by O_2_ diffusion or availability and it reflects the temperature (kinetic) effect on the enzyme nitrogenase. In order to keep the heterocyst micro-oxic the influx of O_2_ must have been precisely controlled.

It is important to note that the heterocyst glycolipid layer forms a gas diffusion barrier that does not discriminate between O_2_ and N_2_^8^. While on the one hand the heterocyst glycolipid layer will limit the rate of O_2_ influx, it must also allow sufficient influx of N_2_ for N_2_ fixation; on the other hand, influx of O_2_ is essential for generating energy through respiratory electron transport when light is unavailable and hence extends the period during which the organism is able to fix N_2_. It is also important to understand that by measuring nitrogenase activity one looks specifically at the processes in the heterocyst, rather than in the vegetative cells. For instance, by measuring the light-independent part of nitrogenase activity, this can be translated to respiration. In the dark, respiration is the only known source of energy for nitrogenase in the heterocyst, and, hence, dark nitrogenase activity can be interpreted as the influx of O_2_ into the heterocyst. When nitrogenase activity is detected one must conclude that the heterocyst is low in O_2_. The residual O_2_ concentration in the heterocyst can be as low as 0.6 µM, which is 0.2% of air saturation^18^ and depends on the *K*_*m*_(O_2_) of the O_2_-scavenging process.

In this investigation, I demonstrate in two ways that *Fischerella* is capable of adjusting the influx of air into the heterocyst. First, the effect of the external O_2_ concentration on nitrogenase activity was measured at a constant temperature. Second, the effect of temperature on nitrogenase activity was measured at a constant external O_2_ concentration. Temperature affects the rate of metabolic processes, including respiration, but has a small effect on the rate of gas influx by diffusion. The gas influx is the combination of O_2_ solubility and diffusion. Whereas diffusion increases with temperature, solubility decreases and the two factors almost compensate each other (*Q*_*10*_ = 1.084)^17^.

**Figure 6:**
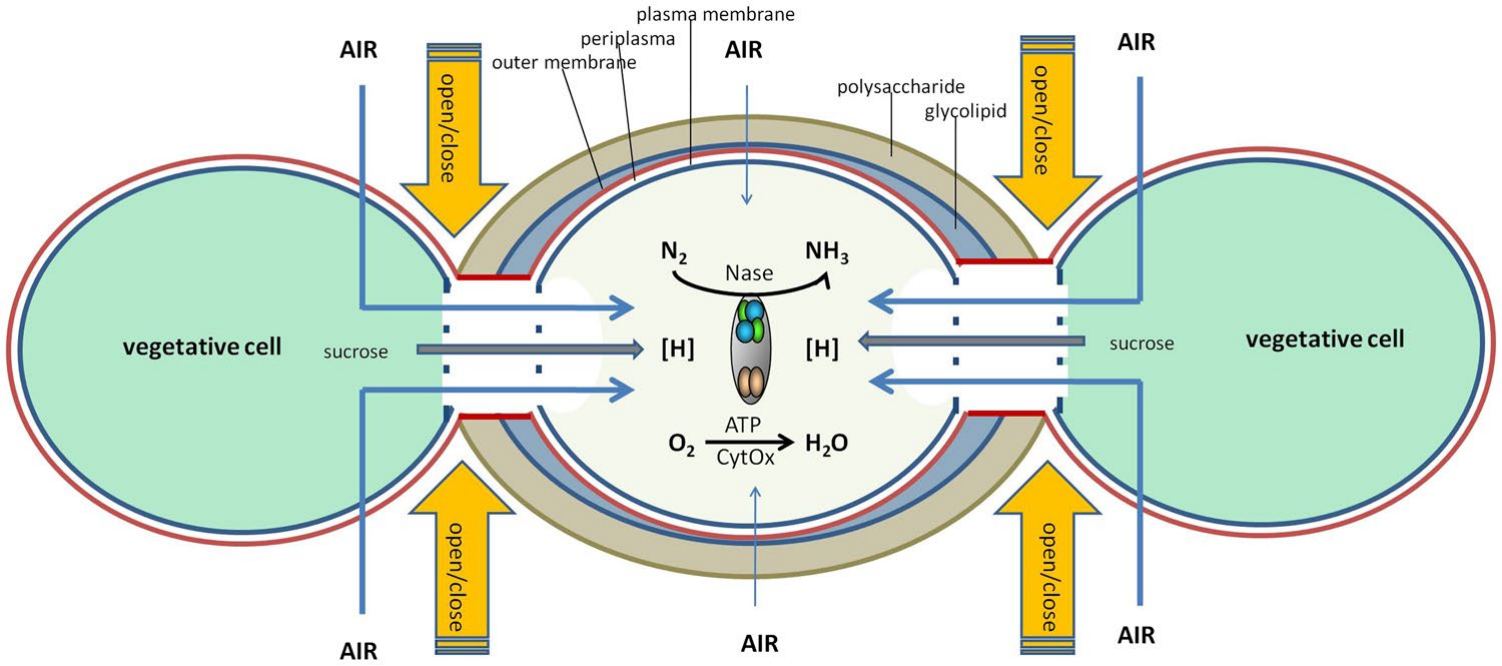
Model of a heterocyst with junctions to a vegetative cell at each side. The model emphasizes the situation for *N*_*d*_ (see Fig. 1 legend). The heterocyst is characterized by an envelope comprising a glycolipid layer, which forms the principal barrier to diffusion of gas (thin arrows), and a polysaccharide layer. The envelope surrounds a cell wall with an outer membrane, peptidoglycan layer, and plasma membrane. Whether or not air enters the heterocysts via the vegetative cells may be regulated by an open/close mechanism (conceptually depicted by orange arrows). O_2_ is respired, producing ATP. N_2_ and O_2_ compete for electrons that are imported as sucrose from the vegetative cells. The pores at the ends of the heterocysts may sometimes contain cyanophycin (polyaspartate-multiarginine polymer). Nase: nitrogenase; CytOx: cytochrome oxidase; [H]: reducing equivalents (NAD(P)H, FdH) derived from the metabolism of sucrose.

I observed that the light independent part of nitrogenase activity increased with temperature in the range from 12–42 °C. The *Q*_*10*_ in the range of 21–39 °C was 2.2. *N*_*d*_ can only increase when respiratory energy generation increases and this is only possible when the O_2_ influx increases. Since these experiments were done under a constant O_2_ concentration in the gas mixture, it was concluded that the heterocyst possesses a mechanism to increase the influx of O_2_. It is unlikely that the heterocyst glycolipid layer possesses the capacity to alter its gas diffusion properties in the short term of the experiment. One possibility that was proposed by Walsby^18^ is that the heterocyst terminal pores might perform this function (analogous to stomata in plant leaves) (Fig. 6). Canini *et al*.^20^ proposed that cyanophycin serves as a plug that closes the neck of the heterocyst connection to the vegetative cell. However, until now hardly anything is known about the physical properties of this insoluble polymer and it is therefore unknown whether this material is gas tight. Nevertheless, these are attractive concepts waiting for experimental proof. Cytoplasmic connections have also been postulated as the site of transport between cells of heterocystous cyanobacteria^11, 19^. The exchange of solutes (such as sucrose) between the vegetative cell and the heterocyst is through the septal junctions (previously named microplasmodesmata) by molecular diffusion^21^. However, although gas molecules could diffuse together with the solutes, they may additionally also diffuse through the cell wall and cytoplasmic membrane which are impermeable for larger molecules such as sucrose.

The *Q*_*10*_ is in the physiological range which means that it is related to the enzyme activity solely and that *N*_*d*_ is not limited (by energy or reducing equivalents) in the temperature range of 21–39 °C. Unless nitrogenase was replaced continuously and instantaneously, it was also unlikely that nitrogenase was inactivated by O_2_, because control experiments during which the cells were brought back to the initial conditions reproduced the original nitrogenase activity. This would require that the gas influx is tightly controlled to ensure that all O_2_ entering the heterocyst is respired. Another remarkable observation was that *N*_*d*_ was less sensitive to temperature than was the light-dependent part of nitrogenase activity, *N*_*m*_. This could indicate that the photosystem is more thermosensitive than the respiratory system. Photosystem II is known as the most thermolabile component that determines the upper thermal tolerance of photosynthesis^22^. It has been shown that the D1 protein degrades faster at high temperature and is not re-synthesized^23^. It is not known whether a similar process affects photosystem I in the heterocyst and makes it more thermosensitive than the respiratory system^24^.

The experiments with different O_2_ concentrations at a constant temperature confirmed the presence of a control mechanism that determines the influx of gas into the heterocyst. Only above 30% O_2_ the supposed influx was a linear function of the external concentration and determined by the diffusion characteristics of the heterocyst envelope. Below 30% O_2_ a dose-response relationship was found. The lower the external concentration of O_2_ the higher the relative influx became. The gas diffusion through the heterocyst envelope would be an intrinsic property of the envelope and would be a linear function of the external O_2_ concentration at any O_2_ level. Subtracting this envelope gas diffusion from the *N*_*d*_ below 30% O_2_, I found an exponential decay of the additional (in excess of the envelope gas diffusion) chlorophyll-specific dark nitrogenase activity, hence O_2_ influx (measured as acetylene reduction), from ~60 µmol mg^−1^ h^−1^ at 0% O_2_ to ~0 µmol mg^−1^ h^−1^ at 30% O_2_ (Fig. 4). Hence, when the terminal pores would serve as a variable gas inlet for the heterocyst they would be ‘open’ at 0% O_2_ and ‘closed’ at 30% O_2_ (Fig. 6).

The lower *N*_*tot*_ with increasing external O_2_ concentration above 30% O_2_ is explained by an increasing demand of reducing equivalents for respiration that are consequently unavailable for the reduction of N_2_. Extrapolation of the data indicated that only at 97% O_2_ *N*_*tot*_/*N*_*d*_ becomes 1, meaning that respiration would theoretically supply all energy for nitrogenase activity. Hence, light remains an important energy source, also perhaps because it generates the electron donor of nitrogenase, reduced ferredoxin, through photosystem I-mediated electron transport^12^.

The measurements reported here were carried out by the acetylene reduction assay. The reduction of acetylene to ethylene is an accepted measure of nitrogenase activity. It should be noted that the reduction of acetylene cannot be converted quantitatively directly into fixation of N_2_. Using the electron stoichiometry, the ratio acetylene reduction to N_2_ fixation is 4:1. If one supposes that the H_2_ produced during N_2_ fixation is recycled by a hydrogenase, the ratio would be 3:1. However, this would only make a difference when reducing equivalents limit N_2_ fixation, which was not the case. Also, the concentration of 10% acetylene does not saturate nitrogenase and the saturation of nitrogenase activity by acetylene *in vivo* also depends on the light intensity^25^. At 10% acetylene, light intensity has only a marginal effect on the saturation of nitrogenase by acetylene and therefore the rates of acetylene reduction in the light response curves do not have to be corrected for this effect. I have not taken into account the solubility and diffusion coefficients of acetylene and ethylene or of N_2_. The diffusion coefficients of acetylene and ethylene may be different from N_2_ (and thus from O_2_). As proposed here, the influx of a gas into the heterocyst through the pores would be equally controlled for acetylene and ethylene as it would be for air. Hence, it is unlikely that the use of the acetylene reduction assay instead of directly measuring N_2_ fixation would have led to different conclusions.

In conclusion, although it is known that heterocystous cyanobacteria can modify their heterocyst glycolipid layer in response to external O_2_ concentration, such acclimation cannot be instantaneous and also depends on the properties of this glycolipid layer which should be genetically determined. For instance, it has been observed that heterocystous cyanobacteria that thrive in colder waters possess a heterocyst envelope with a stronger gas diffusion barrier than those that occur in warmer environments where respiration is faster. The results of this investigation support the idea of Walsby^18^ that heterocysts can also respond instantaneously to O_2_ and temperature as depicted in Fig. 6. It is likely that this is a general property of heterocystous cyanobacteria that contribute to their ecological success.

## Methods

### Organism and growth conditions

*Fischerella* sp. strain MV11 (Culture Collection Yerseke; CCY0604) was obtained from Prof. dr. W.R. Hess, University of Freiburg, Germany, and has been described in detail^26^. This is a true-branching, thermophilic, N_2_-fixing, heterocystous cyanobacterium belonging to the Stigonematales (section V) and was originally isolated from a geothermal site at a hot spring in Costa Rica. The organism was routinely grown at 27 °C in BG11_o_ medium (devoid of any combined nitrogen)^27^ under a 12:12 hour light-dark cycle and 30 µmol m^−2^ s^−1^ warm-white fluorescent light.

### On-line acetylene reduction assay

Nitrogenase activity was monitored using the acetylene reduction assay^28^. Here, I used the on-line set-up as described previously^25, 29^. The set-up allows a highly sensitive measurement of ethylene (0.3 ppbv, parts per billion by volume) at a time resolution of 3–5 seconds and therefore provides almost real-time monitoring of nitrogenase activity. The sensitive and rapid monitoring of ethylene was done using a laser photoacoustic trace gas detector (ETD-300, Sensor Sense, Nijmegen, The Netherlands). A low amount (0.4–0.8 µg chlorophyll *a*) of *Fischerella* culture was gravity filtered on a Whatman GF/F glass fibre filter (47 mm). This low amount of culture prevented self-shading and also excessive ethylene production (which would result in an ‘overload’ of the ETD-300). The filter containing *Fischerella* was put in a custom-made measuring cell^29^. The cell was equipped with a Peltier temperature regulation unit (Supercool DA-075-24-02-00-00, Göteborg, Sweden). The filter was placed on a supporting grid underneath which growth medium was present that ensured that the filter was saturated with medium and that prevented desiccation. The incubation chamber was connected to a continuous flow of gas at 2 L h^−1^ that was led over the filter with *Fischerella*. The gas was a mixture of N_2_, O_2_, CO_2_ and C_2_H_2_, made up by mass flow controllers (Brooks Instruments, Ede, The Netherlands). Acetylene was kept at 10%. CO_2_ was premixed at 0.04% in the N_2_ and O_2_. The concentration of O_2_ was varied as required and the balance was made up with N_2_. O_2_ concentrations ranged from 0–90% (the highest possible concentration since 10% C_2_H_2_ was always present). The gas was washed through water prior to entering the measuring cell. This served to humidify the gas and removed any acetone from the acetylene (which would interfere with the ethylene detection). Gas mixtures (O_2_ and N_2_ with 0.04% CO_2_) were purchased from Hoek-Loos (The Netherlands) and acetylene was obtained from Messer (The Netherlands). The gas mixture contained a very low background level of ethylene of ~10 ppbv due to contamination of the acetylene. Controls were run without *Fischerella* in the incubation chamber. Ethylene recordings of >100 ppbv were taken as positive for ethylene production. The whole set-up was placed in a temperature-controlled climate room. Illumination was provided by a cold-light 250 W halogen lamp and glass fibre light shower (model 460-F, Heinz Walz GmbH, Effeltrich, Germany). A computer program written in Testpoint (Capital Equipment Corporation, New Hampshire, USA) controlled the gas mixtures, light intensity and temperature.

### Light-response curves

At any given temperature and concentration of O_2_ the response of nitrogenase activity to light was measured as follows. Ten different photon irradiances were tested: 0, 1.4, 4, 7.1, 10.9, 15.5, 20.5, 26.5, 43.5 and 68.2 µmol m^−2^ s^−1^. Nitrogenase activity was monitored for 5 minutes at each light level. The measurement started with 15 minutes in the dark, followed by the series of light levels until the highest level tested which saturated nitrogenase activity at all conditions tested (light up), after which the same measurements were made while light levels decreased (light down). This was done to observe any hysteresis effects and to check that the original measurements could be reproduced. The system is robust and measurements were reproducible with high accuracy. A full light response measurement (light up and light down) took 105 minutes after which the computer adjusted the measuring cell to the next condition. The whole experiment ran unattended.

### Temperature experiments

The effect of temperature was measured in two series of experiments. In one series of experiments the temperature was decreased from 27 °C (growth temperature) in steps of 3 °C to 12 °C and in the second experiment the temperature was increased from 27 °C to 60 °C, also in steps of 3 °C. Both experiments were carried out immediately one after the other, using cells from the same stock culture. In the first experiment (decreasing temperature) the filter contained 0.4 µg chlorophyll *a*, and in the second experiment (increasing temperature) it contained 0.7 µg chlorophyll *a*. The nitrogenase parameters at 27 °C from both experiments were similar and hence the data of the two experiments overlapped. In test runs it was demonstrated that cells that were lowered to 18 °C and subsequently brought back to 27 °C, reproduced the original nitrogenase activity and the same was true for cells that were increased to 54 °C and brought back to 27 °C. Below 18 °C (incubations at 15 °C and 12 °C) and above 54 °C (incubations at 57 °C and 60 °C) the cultures were to some extent negatively affected and also possessed low nitrogenase activity.

### Oxygen experiments

The effect of O_2_ was measured in one series of measurements from 0–90% O_2_. The filter contained 0.8 µg chlorophyll *a*. The following O_2_ concentrations were tested and in this order: 0, 2.5, 5, 10, 15, 20, 25, 30, 40, 50, 60, 70, 80, and 90%. The experiment was run at 30 °C. Before starting the experiment the culture was run at 0% O_2_, 20.5 µmol m^−2^ s^−1^ light and 30 °C for 30 minutes by which time a constant rate of nitrogenase activity was achieved. The system did not achieve a fully anoxic state. Previously, it was estimated that the setting 0% O_2_ may in fact represent a level of ~1% O_2_^30^. This is in part because of leakage through the tubing used and the large volume of water (1 L) needed for washing the gas, as well as due to photosynthetic O_2_ production (in the light). Fully anoxic conditions in the dark would not have allowed any activity (since there would not be a possibility to generate energy). At each O_2_ concentration the light response of nitrogenase activity was recorded as described above (light up, light down) at the same 10 light levels as mentioned above for the recording of light-response curves. Test runs were performed before carrying out the final experiment reported here.

### Fitting the light response curves

The light response curves were fitted with the rectangular hyperbola model^31^; equation (5) using the software package Origin. The fitted parameters derived from this model are *N*_*d*_, *N*_*m*_, and α, respectively the light-independent part of the nitrogenase activity (dark activity, which is always present), the maximum light-dependent nitrogenase activity at light saturation, and the slope of the curve. From these fitted parameters, the total maximum nitrogenase activity, *N*_*tot*_, was calculated as the sum of *N*_*d*_ and *N*_*m*_. *N*_*tot*_/*N*_*d*_ is a convenient parameter that is dimensionless and therefore biomass independent (and, hence, refers only to the cells that possess nitrogenase activity, in this case the heterocysts) and describes the contribution of light to nitrogenase activity. *N*_*tot*_/*N*_*d*_ ≥ 1; when this ratio equals 1 no light-dependent nitrogenase activity is present. The light saturation coefficient *I*_*k*_ equals *N*_*m*_/α, and represents the photon irradiance at which 0.5 *N*_*m*_ would have been reached when nitrogenase activity increases linearly with irradiance (*K*_*s*_ in Michaelis-Menten kinetics).

5 $${N}_{I}={N}_{m}(\alpha I/({N}_{m}+\alpha I))+{N}_{d}$$

where *N*_*I*_ is the actual nitrogenase activity at light level *I*.

### Temperature coefficient

The temperature coefficient of nitrogenase activity (*Q*_*10*_) was fitted from the linear fit of the natural log transformed data of *N*_*d*_, *N*_*m*_, and *N*_*tot*_ with temperature^32^ using the software package Origin. Meaningful values (*i.e*. linear fits) of *Q*_*10*_ were obtained in the temperature range 18–39 °C. The temperature coefficient of O_2_ diffusion in the temperature range 18–39 °C was calculated from O_2_ solubility and diffusion coefficients that can be found in the tables provided by the company Unisense and can be downloaded from: http://www.unisense.com/files/PDF/Diverse/Seawater%20&%20Gases%20table.pdf.

### Chlorophyll

Chlorophyll *a* was extracted from the GF/F filters in 2 ml 96% ethanol overnight at room temperature in the dark, immediately after the experiment was finished. The absorption coefficient of 72.3 ml mg^−1^ cm^−1^ was used to calculate the concentration of chlorophyll *a*.
